# Editorial: miRNA Regulatory Pathways in Metazoans. Advances From *in vivo* and *ex vivo* Studies

**DOI:** 10.3389/fgene.2019.00147

**Published:** 2019-03-04

**Authors:** Laurence Amar, Hervé Seitz

**Affiliations:** ^1^Molecular Neuroendocrinology of Food Intake, Institute of Neuroscience Paris-Saclay (NeuroPSI), UMR 9197 CNRS, Université Paris-Sud and Université Paris-Saclay, Orsay, France; ^2^Institut de Génétique Humaine, UMR 9002 CNRS and Université de Montpellier, Montpellier, France

**Keywords:** metazoan microRNAs, intracytoplasmic localization, imprinted microRNA genes, target RNA-directed microRNA degradation, antiviral and proviral microRNAs, small molecule screen, computational pipeline, retrotransposon silencing

The first two microRNAs (miRNAs) were discovered in 1993 and 2000, but the scientific community uncovered their diversity only in 2001 (Lee et al., 1993; Reinhart et al., 2000; Lee and Ambros, 2001; Lagos-Quintana et al., 2001; Lau et al., 2001). Since then, these small regulatory RNAs have attracted much attention, and a rich and diverse literature has described their biogenesis, their mode of action, and the physiological pathways that they control.

Hundreds of miRNAs are expressed from metazoan genomes, and they act as negative regulators for specific target RNAs. Their expression pattern can be more or less specific, with some miRNAs exhibiting high tissue- or developmental stage-specificity, while others tend to be more ubiquitous (Landgraf et al., 2007). Several studies showed that miRNA ablation could have dramatic impacts on development while others demonstrated a role for some miRNAs in the adaptation to various stresses (reviewed in Bartel, 2018). The miRNA pathway is well conserved in most studied metazoans, and some individual miRNAs themselves can be very deeply conserved (Grimson et al., 2008). Together with the dramatic phenotypes observed in some miRNA mutants, these considerations indicate that the miRNA pathway is an essential regulator of gene expression in metazoans. This special issue aims at reviewing the state-of-the-art for several emerging topics in the field, as well as presenting novel results and methodologies regarding miRNA biology.

While the action of miRNAs on their target RNAs is well described from a biochemical point of view (Iwakawa and Tomari, 2015), several intriguing issues remain poorly understood. In their review article, Akgül and Erdoǧan summarize current knowledge regarding the subcellular localization of miRNAs and their effector complex, and potential functional consequences of such spatial regulation of miRNA activity.

Novel miRNA genes have emerged throughout metazoan evolution, and this process is certainly still going on, generating taxon- or species-specific miRNAs besides the most highly conserved miRNAs. In their review article, Malnou et al. discuss a puzzling aspect of miRNA evolution: three taxon-specific genomic loci (respectively rodent-, primate-, and eutherian-specific) cumulate two unusual features. They are composed of many related gene copies that tend to be repeated in tandem, and they are expressed only from one parental allele (either paternal or maternal, depending on the locus). These unusual miRNAs also share another commonality: their expression is markedly (sometimes, almost exclusively) specific to the placenta. While these features appear completely unconnected, their co-occurrence in three distinct genomic loci suggests a causal link: potential explanations are discussed in the Malnou et al. review.

The regulation of miRNA degradation is still a largely open field of research. Recent discoveries showed that miRNA stability can be controlled by some of their target RNAs (either coding or non-coding). In that process, the main determinant for the induction of miRNA degradation seems to be the geometry of paired and unpaired nucleotides between the two RNA molecules. Examples of target-induced miRNA degradation are starting to accumulate, and in their review article, Wightman et al. compiled a very exhaustive description of the known features of such miRNA degradation-inducing interactions.

Among the biological pathways controlled by miRNAs in animals, sensitivity to viral infections constitutes an interesting case: while some miRNAs can play a clear antiviral role, some others are hijacked by viruses to promote viral infection. The complex interplay between mammalian viruses and miRNAs is described in Girardi et al.'s review article.

Besides these four review articles, this special issue features three primary research articles, presenting novel results, and technological advances.

Using a high-throughput screen for small molecules affecting miRNA biogenesis or action, Brustikova et al. identified chemical compounds with a reproductible activating or inhibiting effect. Carefully controlled experiments were used to assess reproducibility and estimate false positive rates, also revisiting previously published compounds.

O'Neill et al. describe a novel computational pipeline for the annotation of small RNAs from Small RNA-Seq libraries. Applying it to actual Small RNA-Seq datasets from melanoma samples, they could identify and annotate small RNAs that are differentially expressed between treatment-sensitive and treatment-resistant cells.

An important issue in miRNA research pertains to the verification of predicted or published miRNA/target regulatory interactions. Because the human miR-128 miRNA had been shown to repress LINE-1 retrotransposons (Hamdorf et al., 2015), because its sequence is perfectly conserved in mouse, and because the miRNA-maturing enzyme Dicer is necessary for LINE-1 repression in murine ES cells (Bodak et al., 2017), it could be expected that miR-128 is also a repressor of LINE-1 in mouse. Yet, Bodak et al. convincingly show that miR-128 does not repress LINE-1 in murine ES cells, within good experimental detection limits. The clear effect of Dicer on LINE-1 expression in murine ES cells therefore does not rely on miR-128.

Overall, the seven articles published in this special issue provide a nice overview of open questions and established knowledge on several key questions in the field of miRNA biology. Technological and conceptual progress is very quick in this dynamical research topic: the presented articles are a great sample of the ongoing research in miRNA biology in animals.

## Author Contributions

All authors listed have made a substantial, direct and intellectual contribution to the work, and approved it for publication.

### Conflict of Interest Statement

The authors declare that the research was conducted in the absence of any commercial or financial relationships that could be construed as a potential conflict of interest.

## References

[B1] Bartel D. P. (2018). Metazoan microRNAs. Cell 173, 20–51. 10.1016/j.cell.2018.03.006 29570994 PMC6091663

[B2] Bodak M. Cirera-Salinas D. Yu J. Ngondo R. P. Ciaudo C. (2017). Dicer, a new regulator of pluripotency exit and LINE-1 elements in mouse embryonic stem cells. FEBS Open Biol. 7, 204–220. 10.1002/2211-5463.12174 28174687 PMC5292673

[B3] Grimson A. Srivastava M. Fahey B. Woodcroft B. J. Chiang H. R. King N.. (2008). Early origins and evolution of microRNAs and Piwi-interacting RNAs in animals. Nature 455, 1193–1197. 10.1038/nature07415 18830242 PMC3837422

[B4] Hamdorf M. Idica A. Zisoulis D. G. Gamelin L. Martin C. Sanders K. J.. (2015). miR-128 represses L1 retrotransposition by binding directly to L1 RNA. Nat. Struct. Mol. Biol. 22, 824–831. 10.1038/nsmb.3090 26367248

[B5] Iwakawa H. O. Tomari Y. (2015). The functions of MicroRNAs: mRNA decay and translational repression. Trends Cell Biol. 25, 651–665. 10.1016/j.tcb.2015.07.011 26437588

[B6] Lagos-Quintana M. Rauhut R. Lendeckel W. Tuschl T. (2001). Identification of novel genes coding for small expressed RNAs. Science 294, 853–858. 10.1126/science.1064921 11679670

[B7] Landgraf P. Rusu M. Sheridan R. Sewer A. Iovino N. Aravin A.. (2007). A mammalian microRNA expression atlas based on small RNA library sequencing. Cell 129, 1401–1414. 10.1016/j.cell.2007.04.040 17604727 PMC2681231

[B8] Lau N. C. Lim L. P. Weinstein E. G. Bartel D. P. (2001). An abundant class of tiny RNAs with probable regulatory roles in *Caenorhabditis elegans*. Science 294, 858–862. 10.1126/science.1065062 11679671

[B9] Lee R. C. Ambros V. (2001). An extensive class of small RNAs in *Caenorhabditis elegans*. Science 294, 862–864. 10.1126/science.1065329 11679672

[B10] Lee R. C. Feinbaum R. L. Ambros V. (1993). The *C. elegans* heterochronic gene *lin-4* encodes small RNAs with antisense complementarity to *lin-14*. Cell 75, 843–854. 10.1016/0092-8674(93)90529-Y 8252621

[B11] Reinhart B. J. Slack F. J. Basson M. Pasquinelli A. E. Bettinger J. C. Rougvie A. E.. (2000). The 21-nucleotide let-7 RNA regulates developmental timing in *Caenorhabditis elegans*. Nature 403, 901–906. 10.1038/35002607 10706289

